# Supplementary material for: Immune Checkpoint and EMT-Related Molecules in Circulating Tumor Cells (CTCs) from Triple Negative Breast Cancer Patients and Their Clinical Impact
Source: Cancers (Basel). 2023 Mar 25;15(7):1974. doi: 10.3390/cancers15071974 (PMC10093450; doi:10.3390/cancers15071974)
Supplement: Supplementary file 1 [file cancers-15-01974-s001.zip › cancers-2289201-supplementary.pdf]

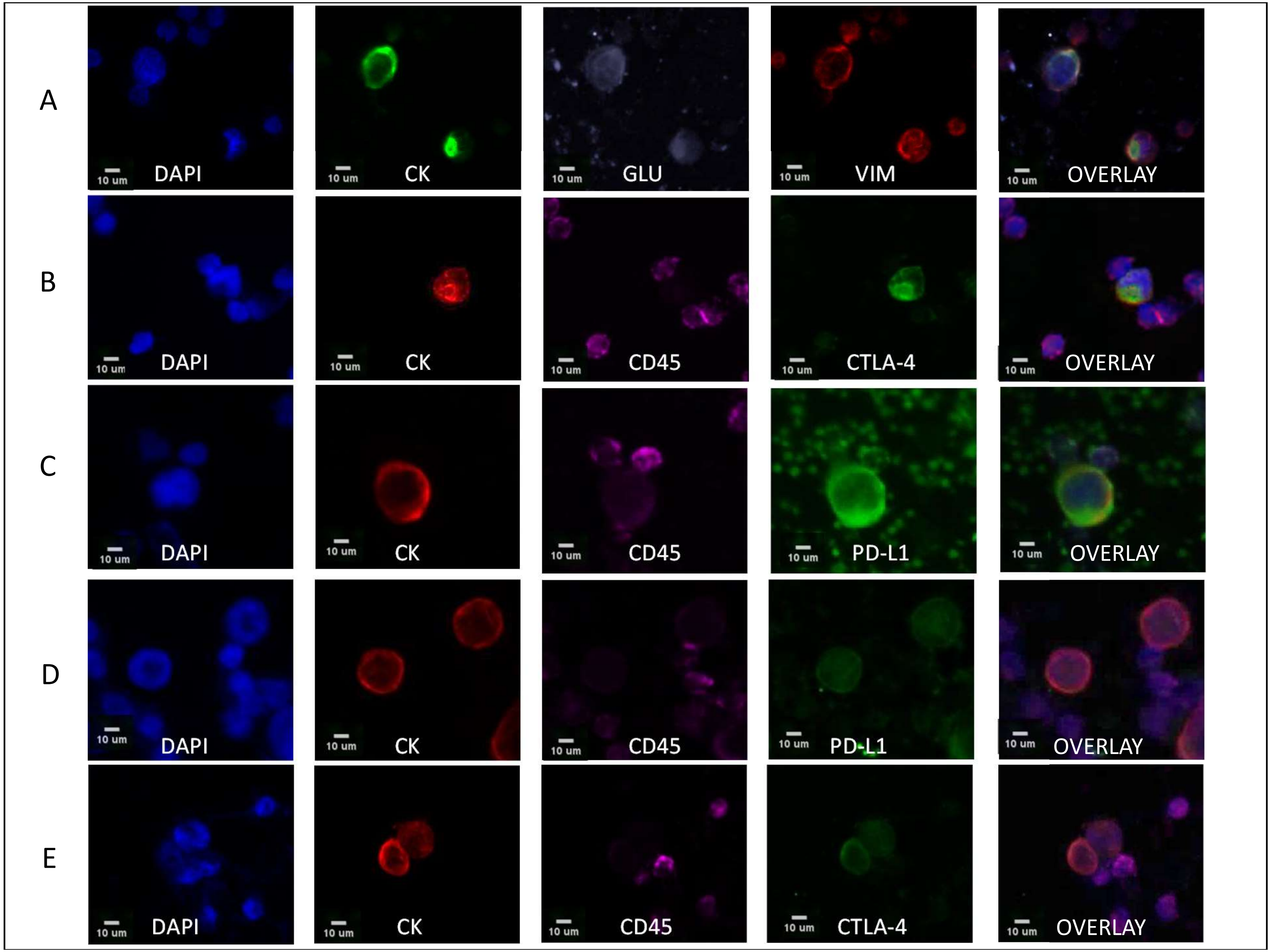

Figure S1. Representative micrographs of the positive controls (MDA-MB-231) of (A) CK/CD45/PDL1, (B) CK/CD45/CTLA-4, and (C) CK/GLU/VIM as well as positive controls (MCF-7) of (D) CK/CD45/PDL1 and (E) CK/CD45/CTLA-4 acquired from VyCAP system.

Table S1. Statistical analysis and *p* values of all the comparisons between different phenotypes in TNBC and luminal patients.

Figure	expression	Test	*p* Values
2b	PD-L1+	Chi-square	0.569
2b	PD-L1-	Chi-square	0.113
2b	CTLA-4+	Chi-square	0.025
2b	CTLA-4-	Chi-square	0.555
2d	PD-L1+	Mann-Whitney	0.150
2d	PD-L1-	Mann-Whitney	0.049
2d	CTLA-4+	Mann-Whitney	0.024
2d	CTLA-4-	Mann-Whitney	0.327
4a	PD-L1+	Chi-square	0.272
4a	PD-L1-	Chi-square	0.790
4a	CTLA-4+	Chi-square	0.189
4a	CTLA-4-	Chi-square	0.755
4b	PD-L1+(early)	Chi-square	0.102
4b	PDL1+(metast.)	Chi-square	0.968
4b	CTLA-4+ (early)	Chi-square	0.424
4b	CTLA-4+ (metast.)	Chi-square	0.172
4c	PD-L1+	Mann-Whitney	0.244
4c	PD-L1-	Mann-Whitney	0.852
4c	CTLA-4+	Mann-Whitney	0.223
4c	CTLA-4-	Mann-Whitney	0.668
4d	PD-L1+(early)	Mann-Whitney	0.551
4d	PDL1+(metast.)	Mann-Whitney	0.179
4d	CTLA-4+ (early)	Mann-Whitney	0.449
4d	CTLA-4+ (metast.)	Mann-Whitney	0.090
5b	GLU+VIM+CK+	Chi-square	0.005
5b	GLU-VIM+CK+	Chi-square	0.842
5b	GLU+VIM-CK+	Chi-square	0.448
5b	GLU-VIM-CK+	Chi-square	0.555
5d	GLU+VIM+CK+	Mann-Whitney	0.020
5d	GLU-VIM+CK+	Mann-Whitney	0.765
5d	GLU+VIM-CK+	Mann-Whitney	0.485
5d	GLU-VIM-CK+	Mann-Whitney	0.604

Table S2. Total number of CTCs, expressing PD-L1, CTLA-4, GLU and VIM in all TNBC and luminal patients

PATIENTS	SUBTYPE	PD-L1+	PD-L1-	CTLA-4+	CTLA-4-	GLU+	GLU-	VIM+	VIM-
1	TNBC	0	0	1	0	2	0	2	0
2	TNBC	0	0	0	0	0	0	0	0
3	TNBC	1	1	0	0	1	6	5	2
4	TNBC	0	0	0	0	0	6	1	5
5	TNBC	1	0	1	3	5	13	10	8
6	TNBC	0	0	0	0	0	0	0	0
7	TNBC	0	0	0	0	4	7	10	1
8	TNBC	1	0	0	0	1	0	0	1
9	TNBC	1	0	0	0	1	5	0	6
10	TNBC	0	0	0	0	0	2	0	2
11	TNBC	0	0	0	0	0	0	0	0
12	TNBC	0	0	1	0	0	2	2	0
13	TNBC	1	0	0	0	1	2	2	1
14	TNBC	1	0	1	0	0	0	0	0
15	TNBC	1	2	1	0	0	0	0	0
16	TNBC	1	0	0	0	12	26	35	3
17	TNBC	0	0	0	0	0	0	0	0
18	TNBC	0	0	0	0	2	0	1	1
19	TNBC	0	2	0	0	0	0	0	0
20	TNBC	0	2	0	0	1	1	1	1
21	TNBC	1	0	0	1	2	1	2	1
22	TNBC	0	0	3	0	1	1	2	0
23	TNBC	0	0	1	0	0	1	1	0
24	TNBC	1	1	0	1	0	0	0	0
25	TNBC	1	0	0	0	0	0	0	0
26	TNBC	0	0	0	0	0	1	1	0
27	TNBC	1	0	2	2	0	0	0	0
28	TNBC	2	0	0	0	0	0	0	0
29	TNBC	0	0	0	0	0	0	0	0
30	TNBC	0	0	0	0	0	0	0	0
31	TNBC	2	0	1	1	2	2	4	0
32	TNBC	0	0	0	0	2	13	12	3
33	TNBC	1	0	1	0	0	0	0	0
34	TNBC	0	1	0	0	0	0	0	0
35	TNBC	0	0	0	0	0	5	3	2
36	TNBC	0	0	1	0	0	0	0	0
37	TNBC	0	0	1	0	2	14	15	1
38	TNBC	0	0	0	0	4	4	2	6
39	TNBC	0	0	0	0	1	3	4	0
40	TNBC	0	0	1	0	1	1	2	0
41	TNBC	2	0	1	1	0	1	1	0
42	TNBC	1	0	0	0	0	0	0	0
43	TNBC	0	0	0	0	1	0	1	0
44	TNBC	0	0	0	0	0	0	0	0
45	TNBC	0	2	0	0	0	0	0	0
46	TNBC	3	3	0	1	0	0	0	0
47	TNBC	0	0	0	0	0	2	2	0
48	TNBC	0	0	0	0	1	3	4	0
49	TNBC	1	1	2	0	0	1	1	0
50	TNBC	0	0	0	0	0	0	0	0
51	TNBC	1	0	1	2	0	0	0	0
52	TNBC	2	0	1	1	3	2	3	2
53	TNBC	0	0	1	0	0	1	1	0
54	TNBC	1	0	1	0	1	1	2	0
55	TNBC	1	0	6	2	1	0	1	0
56	TNBC	1	0	2	0	0	0	0	0
57	TNBC	0	0	0	0	0	1	1	0
58	TNBC	0	0	1	0	0	1	1	0
59	TNBC	3	0	0	0	1	0	0	1
60	TNBC	0	0	1	0	0	0	0	0
61	TNBC	1	0	0	0	0	1	1	0
62	TNBC	0	0	0	0	0	0	0	0
63	TNBC	0	0	0	1	2	0	2	0
64	TNBC	0	0	0	1	0	0	0	0
65	LUMINAL	1	0	0	0	ND	ND	ND	ND
66	LUMINAL	0	0	0	0	ND	ND	ND	ND
67	LUMINAL	0	1	0	1	ND	ND	ND	ND
68	LUMINAL	0	0	0	0	ND	ND	ND	ND
69	LUMINAL	1	0	1	0	ND	ND	ND	ND
70	LUMINAL	0	0	0	0	ND	ND	ND	ND
71	LUMINAL	1	1	0	0	ND	ND	ND	ND
72	LUMINAL	0	0	0	0	ND	ND	ND	ND
73	LUMINAL	0	0	0	1	ND	ND	ND	ND
74	LUMINAL	1	0	0	0	ND	ND	ND	ND
75	LUMINAL	0	1	0	0	ND	ND	ND	ND
76	LUMINAL	1	0	0	1	ND	ND	ND	ND
77	LUMINAL	0	1	0	1	ND	ND	ND	ND
78	LUMINAL	1	0	0	0	ND	ND	ND	ND
79	LUMINAL	0	0	0	0	ND	ND	ND	ND
80	LUMINAL	1	0	0	0	ND	ND	ND	ND
81	LUMINAL	0	3	0	0	ND	ND	ND	ND
82	LUMINAL	0	0	0	0	ND	ND	ND	ND
83	LUMINAL	2	0	2	0	ND	ND	ND	ND
84	LUMINAL	0	0	1	0	ND	ND	ND	ND
85	LUMINAL	0	0	0	0	ND	ND	ND	ND
86	LUMINAL	0	0	2	0	ND	ND	ND	ND
87	LUMINAL	0	0	1	0	ND	ND	ND	ND
88	LUMINAL	0	0	0	0	ND	ND	ND	ND
89	LUMINAL	0	0	0	0	ND	ND	ND	ND
90	LUMINAL	0	0	2	0	ND	ND	ND	ND
91	LUMINAL	0	0	0	0	ND	ND	ND	ND
92	LUMINAL	0	0	0	0	ND	ND	ND	ND
93	LUMINAL	0	0	0	0	ND	ND	ND	ND
94	LUMINAL	0	0	0	0	ND	ND	ND	ND
95	LUMINAL	2	0	1	1	ND	ND	ND	ND
